# The Relationship between Trait Impulsivity and Everyday Executive Functions among Patients with Type 2 Diabetes Mellitus: The Mediating Effect of Negative Emotions

**DOI:** 10.1155/2023/5224654

**Published:** 2023-08-22

**Authors:** Na Liu, Chun-Ni Heng, Yi Cui, Ling Li, Yan-Xue Guo, Qin Liu, Bao-Hua Cao, Di Wu, Yin-Ling Zhang

**Affiliations:** ^1^Department of Nursing, Air Force Medical University, Xi'an, China; ^2^Department of Endocrinology, The Second Affiliated Hospital of Air Force Medical University, Xi'an, China; ^3^Department of Endocrinology, The Second Medical Center of Chinese PLA General Hospital, Beijing, China; ^4^Department of Military Medical Psychology, Air Force Medical University, Xi'an, China

## Abstract

**Background:**

In recent years, the incidence of type 2 diabetes mellitus (T2DM) has dramatically increased, imposing a heavy financial burden on society and individuals. The most cost-effective way to control diabetes is diabetes self-management, which depends on patients' executive functions (EFs). However, the level of EFs among patients with T2DM varies greatly. In addition to diabetes-related factors contributing to a decline in EFs, trait impulsivity as a relatively stable personality trait may explicate individual differences in EFs. The objective of this study was to verify the mediating effect of negative emotions on the relationship between trait impulsivity and EFs among patients with T2DM in China.

**Methods:**

A total of 305 patients with T2DM were enrolled consecutively from the endocrinology departments of three tertiary hospitals in China using convenience sampling. The participants completed the Sociodemographic Questionnaire, Mini-Mental State Examination (MMSE), Barratt Impulsiveness Scale-Brief (BIS-Brief), Depression Anxiety and Stress Scales with 21 items (DASS-21), and Behavior Rating Inventory of Executive Function-Adult (BRIEF-A) version. A structural equation modeling was used to verify the mediating effect of negative emotions on the relationship between trait impulsivity and EFs.

**Results:**

A total of 32.46% of the participants experienced at least one aspect of daily EF decline. The mediating effect of trait impulsivity on the Behavioral Regulation Index (BRI) of EFs through negative emotions was significant, accounting for 29.57% of the total effect. The mediating effect of trait impulsivity on the Metacognitive Index (MI) of EFs through negative emotions was significant, accounting for 31.67% of the total effect.

**Conclusions:**

Trait impulsivity can positively predict EF decline, which can be alleviated by improving the negative emotions of patients with T2DM. Future research exploring interventions to improve the EFs of patients with T2DM should therefore consider their trait impulsivity and negative emotions.

## 1. Introduction

Diabetes mellitus (DM) has been the fastest-growing global health issue of the 21st century, seriously endangering human health. As reported by the International Diabetes Federation (IDF) in 2021, diabetes affects approximately 537 million adults worldwide. It is predicted that approximately 783 million individuals will live with diabetes by 2045 [1]. Among them, more than 90% will be patients with type 2 diabetes mellitus (T2DM) [2]. China has 140.9 million patients with diabetes—the largest number of all countries globally. There is no effective radical treatment for diabetes at present, and lifelong diabetes treatment or management must be undertaken for patients. Diabetes management involves a series of health behaviors, including strict diet control, regular exercise, timely medication administration, regular blood glucose level monitoring, and regular follow-ups at the hospital, which can be effectively carried out by the patients themselves. Such diabetes self-management mainly depends on patients' executive functions (EFs), which are a group of interrelated (though possibly distinct) control processes involved in directing and monitoring cognition, behavior, and emotion [3]. The decline in EFs is bound to affect individuals' daily behaviors, limiting their ability to adapt to environmental requirements or changes [4]. For patients with T2DM, the decline in EFs can negatively affect their diabetes self-management behaviors, which may further aggravate their symptoms. Therefore, the effective treatment of diabetes depends on the successful improvement in EFs.

Many factors have been confirmed to affect the EFs of patients with T2DM. In addition to diabetes-related factors, including hyperglycemia, hypoglycemia, and many pathophysiological indexes, personality characteristics may also be important influencing factors [4, 5]. Among them, impulsive personality has attracted attention, as it has been shown to be significantly associated with T2DM and to negatively impact its management [6, 7]. Impulsivity results in rapid, unplanned responses to internal and environmental stimuli regardless of the consequences [8, 9]. Impulsivity has various expressions, including trait-like personality characteristics, specific behaviors, and cognitive performance [10–12]. In this study, we analyzed impulsivity as a relatively stable personality trait. Trait impulsivity has been linked to EFs in several previous studies. Pietrzak et al. [13] found that trait impulsivity was significantly positively correlated with poorer performance on several EF tests in healthy young adults. Cuvillier and Bayard [14] found that trait impulsivity had a significantly positive ability to predict EF decline in daily life among elderly individuals. In addition, a significantly positive correlation between trait impulsivity and executive dysfunction was also observed in patients with Huntington's disease [15]. Although the literature relating impulsivity to EFs among patients with diabetes is limited, given the respective close associations of trait impulsivity and EFs with diabetes and its management, trait impulsivity may be useful in predicting EF decline among patients with T2DM.

Additionally, cognitive load theory proposes that negative emotions can increase the activation of the widespread connection network of emotion-related thoughts that, in turn, consume cognitive resources and impair individual performance in cognitive activities, thereby decreasing EFs [16]. Several previous studies have confirmed the significant correlation between negative emotions and EFs. Chow et al. [17] found that depression was associated with worse cognition and higher dementia risk. Lin et al. [18] found that self-reported anxiety predicted impairments in EFs. Ajilchi et al. [19] found that the EFs of students with depression, anxiety, and stress symptoms were significantly worse than those of healthy students. Among patients with diabetes, a relationship between negative emotions and impaired EFs was also found. A recent meta-analysis suggested that depression is a significant influencing factor of diabetes-related EF decline [4]. Negative emotions (e.g., anxiety, depression, and stress) are common among patients [20–22]. In particular, the COVID-19 pandemic exacerbated the negative emotions of patients with diabetes. As suggested by several studies, after the start of the COVID-19 pandemic, more negative emotions were reported among patients with diabetes due to fear of infection, risk of symptoms requiring hospitalization, and concerns over the lack of therapeutic medications [23, 24]. Research has found that negative emotions are more likely to occur among individuals with a highly impulsive personality. A prospective study conducted in a large, nonclinical population suggested that high impulsivity could be a predictor of depression among healthy adults [25]. In addition, a cross-sectional study found significantly positive correlations between trait impulsivity and depression, anxiety, and stress [26]. Thus, negative emotions may play a crucial role in the relationship between trait impulsivity and EFs.

Previous studies have found that trait impulsivity, negative emotions, and EFs are significantly correlated with each other. However, no study has investigated the relationship among these three factors among patients with T2DM in the same study. In the current study, we used structural equation modeling (SEM) to examine the relationships among the three and verify the mediating effect of negative emotions on the relationship between trait impulsivity and EFs. SEM is a set of multivariate statistical techniques used to measure latent (unobserved) variables with sets of observed indicators and then to analyze the structural relationships between the latent variables or between observed covariates and latent variables [27–29]. Based on previous studies, we propose two hypotheses: (H1) trait impulsivity positively predicts EF decline among patients with T2DM, and (H2) negative emotions such as depression, anxiety, and stress mediate the relationship between trait impulsivity and EFs.

## 2. Materials and Methods

### 2.1. Participants and Procedures

This study was a cross-sectional descriptive study that enrolled patients with T2DM from the departments of endocrinology of three tertiary hospitals in China using convenience sampling (the researcher announces the study and participants self-select if they wish to participate), which is a form of nonprobabilistic sampling commonly used in population and clinical studies [30]. And the type of sample was nonrandom probability sample. The inclusion criteria were as follows: (1) T2DM patients who met the 1999 WHO diagnostic criteria for diabetes, (2) course of disease ≥ 6 months, (3) age ≥ 18 years old, (4) good verbal communication and understanding skills, (5) MMSE score ≥ 26, and (6) informed consent and willingness to participate in the study. Patients who (1) had a history of cerebrovascular disease or other central nervous system injury and (2) had difficulty completing the questionnaire were excluded. According to the Kendal estimation principle, the sample size should be 5-10 times the total number of independent variables [31]. In the current study, there were 29 total independent variables. If the dropout rate of the subjects is controlled to within 20%, the sample size should be more than [29 × 5 × (1 + 20%)] = 174. In addition, Mueller [32] suggested that the sample size of the structural equation model should be at least 200. Ultimately, 305 subjects were recruited to participate in the study.

Data collection was conducted face-to-face. First, the researchers explained the purpose and significance of the study to the participants using uniform instructions and obtained written informed consent from them. Then, the cognitive screening of the participants was competed in a quiet and comfortable environment. It took them 5-10 minutes to finish the MMSE scale in question-answer form. During this process, the researchers scored the participants' answers according to the instructions. Finally, the participants completed the remaining questionnaires by themselves, which took 15-20 minutes. After receiving the questionnaires, the researchers scrutinized all items to ensure that there were no omissions or errors. The participants could opt out of the study at any time during the survey. This study was approved by the Ethics Committee of Air Force Medical University (no. 202206-02).

### 2.2. Measures

#### 2.2.1. Sociodemographic Questionnaire

The questionnaire was self-designed by the investigators for the purpose of the study. The questionnaire included age, sex, education level, monthly family income, duration of diabetes, history of hypoglycemia in the past year, diabetic complications, treatment method, HbA1c (%), and BMI.

#### 2.2.2. Mini-Mental State Examination (MMSE)

The MMSE remains the most commonly used screening instrument for global cognition [33]. It mainly assesses the following aspects: orientation, attention, language, memory, and EFs. The scale has a total score of 30 points. Previous studies have suggested that scores of the scale are affected by age, gender, and education level [34]. Different studies obtained different optimal cutoff points for dementia screening, ranging from 19 to 26 in the Chinese population [34–37]. Thus, we considered MMSE scores ≥ 26 points to indicate a normal cognitive level in our study [35, 37].

#### 2.2.3. Barratt Impulsiveness Scale-Brief (BIS-Brief)

BIS-Brief [38] was used to measure the trait impulsivity of participants in this study. The BIS-Brief was simplified from the 30-item BIS-11, the most widely used tool globally to measure trait impulsivity. The BIS-Brief contains 8 items that form the 2 factors of poor self-regulation and impulsive behavior [39, 40]. Each factor consists of 4 items. All items were rated on a four-point Likert-type scale from 1 to 4. Items 1, 4, 5, and 6 are reverse-scored questions. A higher total score indicates greater trait impulsivity. Steinberg et al. [38] verified that the BIS-Brief has reliability estimates and construct validity similar to those of the BIS-11, making the BIS-Brief ideal for large epidemiological surveys on account of the least burden on participants [41]. Cronbach's *α* for the total scale was 0.754, and for each dimension, it ranged from 0.754 to 0.848 in this study.

#### 2.2.4. Depression Anxiety and Stress Scales with 21 Items (DASS-21)

The DASS-21 was used to evaluate the participants' negative emotions [42]. The 21 items describe a person's negative emotional experience or corresponding physiological reaction over the last week. The participants made a judgment on the degree to which the description of each item corresponded to their own situation. All items were rated on a four-point Likert-type scale ranging from 0 to 3. The 21 items form the three subscales depression, anxiety, and stress. Each subscale includes seven items. The scores for the three subscales and the total scores for this questionnaire were calculated, with higher scores reflecting greater negative emotions. Cronbach's *α* for the total scale was 0.926, and for each subscale, it ranged from 0.763 to 0.838 in this study.

#### 2.2.5. Behavior Rating Inventory of Executive Function-Adult (BRIEF-A) Version

BRIEF-A is a standardized self-report questionnaire that measures individuals' everyday EFs by assessing the behaviors associated with EF deficits in everyday life in adults [43]. The behaviors are related to abilities to control impulses (the “inhibit” subscale), solve problems flexibly (the “shift” subscale), modulate emotional responses appropriately (the “emotional control” subscale), monitor one's own behavior (the “self-monitor” subscale), begin a task or activity (the “initiate” subscale), hold information in mind (the “working memory” subscale), set goals or perform tasks in a systematic manner (the “plan/organize” subscale), check work (the “task monitor” subscale), and keep materials orderly (the “organization of materials” subscale). The nine subscales form two indexes: the Behavioral Regulation Index (BRI), including four clinical subscales (inhibit, shift, emotional control, and self-monitor), and the Metacognitive Index (MI), including five subscales (initiate, working memory, plan/organize, task monitor, and organization of materials). Finally, the Global Executive Composite (GEC) score comprises all of the subscales. The BRIEF-A contains 75 items, of which five infrequency items are designated to detect atypical responses, and 70 items assess EFs. All items were rated on a three-point scale ranging from 1 to 3. The total points of each subscale and index were transformed into age-appropriate *t* scores based on normative data [44]. Higher *t* scores reflect greater impairments in EFs. It has been proven that the BRIEF-A has good validity and reliability in China [44]. Cronbach's *α* for the total scale was 0.973, and for each subscale, it ranged from 0.747 to 0.882 in this study.

### 2.3. Statistical Analyses

The data were analyzed by IBM SPSS Statistics version 23.0. We performed descriptive statistical analyses for sociodemographic characteristics, trait impulsivity, negative emotions, and EFs. Trait impulsivity, negative emotions, and EFs are all continuous variables. We compared the participants' EF *t* scores with the normative data using a one-sample *t* test. An independent *t* test or one-way ANOVA was performed to compare EFs across sociodemographic subgroups. In addition, Pearson's correlation coefficient (*r*) was used to explore the correlations among the study variables. A *p* level of less than 0.05 was considered statistically significant.

The structural equation modeling was established using *Mplus version 8.3*. We took trait impulsivity as an independent variable, negative emotions as a mediating variable, and the two indexes (BRI and MI) of EFs as dependent variables. The measurement model was evaluated by the maximum likelihood method. Bootstrapping was used to conduct the mediating effect test and to estimate the 95% confidence interval (95% CI) by repeated sampling 1000 times [45]. The indirect effect was considered to reach a significance level if the 95% CI for the effect did not include 0.

## 3. Results

### 3.1. Descriptive Analysis of EF Scores among the Participants and Comparison with Normative Data

Table 1 shows the results of the descriptive analysis of the EF scores among the patients with T2DM. The mean *t* scores for the nine clinical subscales ranging from high to low were emotional control (54.33 ± 10.59), working memory (53.70 ± 10.92), shift (52.98 ± 9.78), task monitor (51.21 ± 9.77), inhibit (51.20 ± 9.69), initiate (49.76 ± 10.60), plan/organize (49.30 ± 10.56), self-monitor (48.73 ± 10.74), and organization of materials (47.92 ± 9.26). The highest mean *t* score of the two indexes was for BRI (52.74 ± 11.35), followed by MI (50.30 ± 10.87). Among the nine clinical subscales, the percentage of participants with a score of 65 or greater, which was considered “abnormally elevated,” and ranging from high to low were working memory (18.69%), emotional control (15.74%), shift (14.43%), plan/organize (13.11%), task monitor (10.82%), initiate (10.16%), inhibit (10.16%), self-monitor (10.49%), and organization of materials (6.56%).

Table 2 shows the prevalence of clinically elevated BRIEF-A scales. The results reflected that 32.46% of the participants had at least one clinically elevated scale, 23.28% demonstrated clinically elevated scores in multiple domains, and 67.54% had no clinically elevated scores.

**Figure 1:**
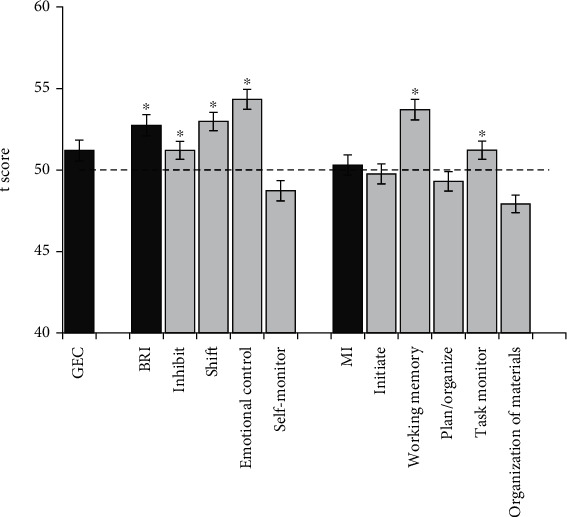
Comparison between the mean *t* scores for each index and clinical scale with the normative mean (*t* scores = 50). The dashed line represents the normative mean (*t* score = 50). Error bars represent the standard error of the mean. Statistically significant effects (*p* < 0.05) are denoted by an asterisk.

Figure 1 shows the results of the comparative analysis between the mean *t* scores for each of the BRIEF-A scales among the participants and the normative mean (*t* scores = 50). The results reflected that the mean *t* scores for BRI were significantly elevated in comparison with the normative data (*t* = 4.21, *p* < 0.001, *d* = 0.24). However, the mean *t* scores for MI were not significantly elevated (*t* = 0.59, *p* = 0.554, *d* = 0.03). Further analyses showed a statistically significant increase in scores on the inhibit, shift, and emotional control scales, which mainly contributed to the elevations in BRI composite scores (*t* > 2.01, *p* < 0.05, *d* > 0.11 in all instances). The mean *t* scores for the task monitor and working memory scales, which contributed to the MI composite score, also significantly increased (*t* > 2.17, *p* < 0.04, *d* > 0.12 in both instances). The mean *t* scores for GEC and the other remaining subscales were not significantly elevated compared to the standard data (*p* > 0.05).

**Figure 2:**
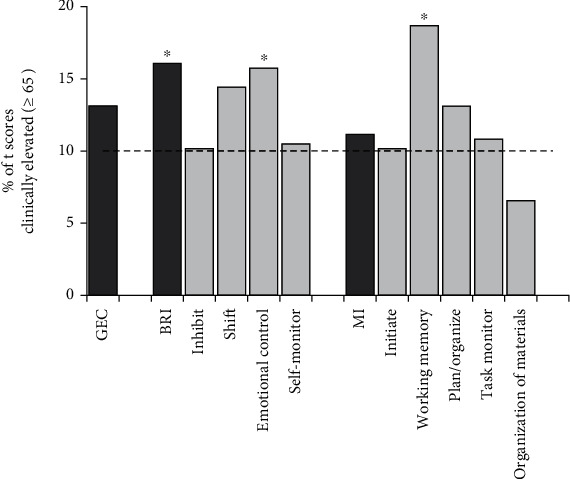
Comparison between the frequency of participants who had a score of 65 or greater on each respective index/scale with normative data. The dashed line reflects the expected frequency (8–10%) based on normative data from the BRIEF-A. Statistically significant effects (*p* < 0.05) are denoted by an asterisk.

Although the scores for some aspects of EFs increased significantly in our study, the magnitude of the increase was modest. For instance, the mean score of the emotional control scale with the largest increase was 54.33, which was only 4 points above the standard mean (*M* = 50, SD = 10). Therefore, we further investigated the clinical significance of the elevations observed in our study. We compared the percentage of abnormally elevated *t* scores (*t* scores ≥ 65) of each subscale and index in our study to the expected percentage (approximately 8-10%) based on standard data from the BRIEF-A. The results are presented in Figure 2. Briefly, there was a statistically significant increase in the percentage of “abnormally elevated” scores for BRI and two clinical subscales (i.e., working memory and emotional control) (*χ*^2^ > 4.75, *p* < 0.03 in all instances) in our study. Among them, the working memory scale had the highest percentage (18.69%) of abnormally elevated scores, which was more than twice as high as the expected percentage (percentage = 8.8% across all age and sex groups) based on normative data from the BRIEF-A (*χ*^2^ = 12.43, *p* < 0.001).

### 3.2. Sociodemographic Characteristics and Comparisons of EFs of the Study Sample

Table 3 presents the sociodemographic characteristics and the results of the comparisons of EFs of the study sample. The average age of the 305 subjects was 52.52 ± 10.80 years (range = 20 − 75). A total of 80.98% of the subjects were male. Approximately 47.54% of the participants had a course of diabetes of 10 years or more. More than half of the patients had one or more diabetic complications, and 44.26% of the patients reported one or more episodes of hypoglycemia in the past year. The vast majority of the patients were treated with oral hypoglycemic drugs, insulin injections, or a combination of both. Only 34.10% of the patients achieved glycemic control targets (HbA1c < 7%). Approximately 63% of the patients were overweight.

For EFs among the patients with T2DM, statistically significant differences were observed in education level (*F* = 5.193, *p* = 0.002), monthly family income (*F* = 5.937, *p* = 0.003), duration of diabetes (*t* = −2.465, *p* = 0.014), treatment method (*F* = 4.224, *p* = 0.006), and HbA1c (%) (*t* = −1.991, *p* = 0.047).

### 3.3. Correlation Analysis of Trait Impulsivity, Negative Emotions, and EFs

Table 4 shows the Pearson correlation analysis of trait impulsivity, negative emotions, and EFs. The results showed that trait impulsivity was significantly positively correlated with negative emotions (*r* = 0.396, *p* < 0.01) and EF scores (*r* = 0.552, *p* < 0.01). Negative emotions were significantly positively correlated with EF scores (*r* = 0.663, *p* < 0.01).

### 3.4. Mediating Analysis

When taking into account all variables (including covariates), the results of mediating analysis suggest that all of the fit indexes were within the acceptable range, specifically the chi-square test (*χ*^2^) = 261.516, degree of freedom (df) = 130, *χ*^2^/df = 2.01 < 4, root mean square error of approximation (RMSEA) = 0.058 < 0.08, comparative fit index (CFI) = 0.961 > 0.95, Tucker-Lewis index (TLI) = 0.952 > 0.95, CFI/TLI = 1.01 > 0.9, and standardized root mean square residual (SRMR) = 0.066 < 0.08 [46].

**Figure 3:**
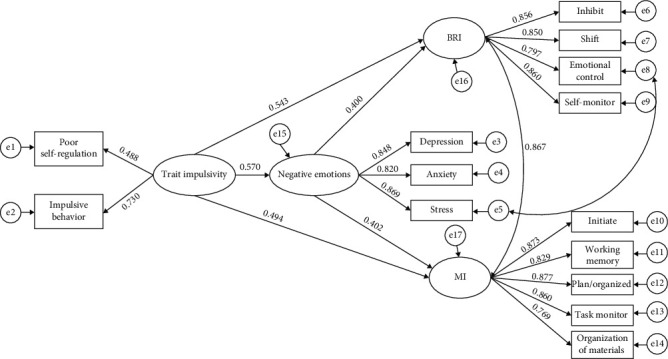
Structural equation modeling of the mediating effect of negative emotions on the relationships between trait impulsivity and BRI and MI of EFs (*n* = 305).

Table 5 shows the 95% CI of each path, and Figure 3 presents the structural equation model. The results showed that negative emotions partially mediated the relationship between trait impulsivity and BRI and MI. The mediating effect of negative emotions on BRI and MI accounted for 29.57% and 31.67% of the total effect, respectively.

## 4. Discussion

The current study validated the mediating effect of negative emotions on the relationship between trait impulsivity and everyday EFs among patients with T2DM. The findings provide a scientific theoretical basis and a new idea for developing strategies to alleviate the decline in EFs among patients with T2DM. To our knowledge, this is the first study to systematically explore the mechanisms underlying the influence of impulsive personality traits and negative emotions on everyday EFs among patients with T2DM, and we focused on patients with normal global cognitive function.

The prevalence of everyday EF impairment was high among the patients with T2DM. In the current study, nearly one-third of the participants had at least one aspect of daily EF decline. Significantly, all of the participants had normal global cognition according to MMSE scores. This finding suggested that impaired EFs may occur in patients with T2DM at the stage of normal global cognitive function and was consistent with a previous study conducted by resting-state functional magnetic resonance imaging and performance-based measures of EFs [47]. However, to our knowledge, this is the first study to investigate everyday EFs using the BRIEF-A in patients with T2DM, particularly cognitively normal individuals. According to the results of the subsequent analysis, significantly elevated scores on the BRI composite were observed. With regard to specific aspects, significantly elevated scores on five clinical subscales, emotional control, working memory, shift, task monitor, and inhibit scales, were observed. Among them, the working memory scale suggested the largest effect size, with 18.69% of the participants having scores in the “abnormally elevated” range (*t* scores ≥ 65) [43]. This finding suggests that the impairment of daily EFs in patients with T2DM is manifested in multiple aspects. Therefore, to effectively hamper the progression of impaired EFs to a more severe form of cognitive impairment so that patients can carry out their daily functional and social activities properly [48], attention should be given to the daily EFs of patients with T2DM as early as possible.

The present study also suggested that patients with a lower education level, lower monthly income, longer duration of diabetes, more complex treatment method, and higher HbA1c had more impaired EFs. Education plays a crucial role in EFs among patients with T2DM [49]. Early education can stimulate and improve the functional efficiency of the cognitive system [50]. Such functional changes may be greater with higher education levels, as a result alleviating the decline in EFs. Conversely, a lower education level predicts more severe impairment of EFs. On the other hand, low-educated patients with T2DM may have poor medical treatment compliance, thus resulting in poor control of diabetes that, in turn, leads to neurocognitive impairment [49]. Similarly, patients with lower monthly income cannot receive timely and effective treatment, which leads to detrimental T2DM effects on EFs. The longer the course of diabetes was, the greater the impaired EFs. Patients with a more complex treatment method and a higher HbA1c tend to have poor control of their diabetes; thus, their EFs are impaired. Impaired EFs both increase the likelihood of T2DM development and are exacerbated by the presence of T2DM [51]. However, regardless of the direction of influence between these two, prevention and alleviation of the impairment of EFs is of great significance in the treatment of diabetes.

Negative emotions mediated the relationships of trait impulsivity with BRI and MI. Namely, trait impulsivity not only directly positively predicted the decline in BRI and MI but also indirectly predicted the decline in BRI and MI through negative emotions. BRI and MI are two indexes that broadly measure EFs. The BRI mainly assesses a person's ability to appropriately regulate behavior and emotional responses. MI mainly assesses a person's ability to actively organize information and solve various problems encountered in the process of completing complex tasks [43]. Cuvillier and Bayard [14] proposed that trait impulsivity, as a relatively stable personality trait, may explicate individual differences in EFs. Individuals with higher impulsivity must exert more cognitive effort to overcome the trait impulsivity that influences impulsive responding, which may lead to a decline in daily EFs [15]. Granö [25] claimed that trait impulsivity affects how individuals adapt to the demands or possibilities of the environment, e.g., stressful life events; thus, impulsive individuals are more prone to experience adverse life events that, in turn, act as triggers for negative emotions. Patients with impulsivity issues are more likely to be affected and have more negative emotions. Negative emotions increase patients' attention preferences for those stressful life events, in turn depleting cognitive resources and interfering with effective behavior and problem solving, thus impairing EFs [16, 52]. Therefore, health care workers should pay more attention to patients with T2DM who have higher impulsivity and screen and intervene in their negative emotions in time to prevent their EFs from declining.

## 5. Strengths and Limitations of the Study

The strength of this study is that we revealed that negative emotions such as depression, anxiety, and stress mediate the relationship between trait impulsivity and EFs, which provides a theoretical basis for future intervention studies to improve the daily EFs of T2DM patients. Furthermore, the participants of this study were from multiple centers, increasing the credibility of the study conclusions. However, since it is a cross-sectional study, there is a limitation of the causal effect. Longitudinal studies are needed to better elucidate the mediating effect of negative emotions. In addition, since this study adopted the questionnaire measurement method to conduct the research, there may be subjective biases that is another limitation of this study. Objective research tools, such as behavioral tasks, should be used in combination in the next step. Moreover, convenience sampling was utilized, which may not represent the total population. Because this study included only three tertiary hospitals in China, the outcomes of this study must be regarded with caution. The emerging model developed from this study should be evaluated with bigger sample size in the future.

## 6. Conclusions

In conclusion, this paper presents the first evidence of the mediating effect of negative emotions on the relationships between trait impulsivity and everyday EFs among patients with T2DM. We found that trait impulsivity can positively predict EF decline, which can be alleviated by improving the negative emotions of patients with T2DM. Future research exploring interventions to improve EFs in patients with T2DM should therefore consider their trait impulsivity and negative emotions.

## Figures and Tables

**Table 1:** Descriptive analysis of EF scores among the participants (*n* = 305).

	*t* scores (*M* ± SD)	Range	*t* ≥ 65 (*n*, %)
Inhibit	51.20 ± 9.69	37-87	31 (10.16%)
Shift	52.98 ± 9.78	39-82	44 (14.43%)
Emotional control	54.33 ± 10.59	38-97	48 (15.74%)
Self-monitor	48.73 ± 10.74	37-94	32 (10.49%)
BRI	52.74 ± 11.35	36-96	49 (16.07%)
Initiate	49.76 ± 10.60	37-92	31 (10.16%)
Working memory	53.70 ± 10.92	39-91	57 (18.69%)
Plan/organize	49.30 ± 10.56	38-87	40 (13.11%)
Task monitor	51.21 ± 9.77	37-83	33 (10.82%)
Organization of materials	47.92 ± 9.26	36-89	20 (6.56%)
MI	50.30 ± 10.87	36-93	34 (11.15%)
GEC	51.19 ± 11.23	35-97	40 (13.11%)

BRI: Behavioral Regulation Index; MI: Metacognitive Index; GEC: Global Executive Composite.

**Table 2:** Prevalence of BRIEF-A scale elevations (*n* = 305).

Number of elevated scales	Frequency	Percentage (%)	Cumulative percentage (%)
0	206	67.54	67.54
1	28	9.18	76.72
2	24	7.87	84.59
3	12	3.93	88.52
4	6	1.97	90.49
5	9	2.95	93.44
6	5	1.64	95.08
7	3	0.98	96.06
8	7	2.30	98.36
9	5	1.64	100

BRIEF-A: Behavior Rating Inventory of Executive Function-Adult.

**Table 3:** Sociodemographic characteristics and comparisons of EFs of the study sample (*n* = 305).

Variables	*N* (%)	*t* scores of GEC (*M* ± SD)	*t*/*F*	*p*
Age			*F* = 0.316	0.129
<45	66 (21.64)	51.11 ± 9.98		
45~60	149 (48.85)	50.77 ± 10.56		
≥60	90 (29.51)	51.96 ± 13.12		
Gender			*t* = −0.662	0.509
Male	247 (80.98)	50.98 ± 11.04		
Female	58 (19.02)	52.07 ± 12.08		
Education level			*F* = 5.193	0.002
Junior high school and below	60 (19.67)	52.83 ± 12.19		
High school	97 (31.80)	53.86 ± 12.65		
Junior college	67 (21.97)	49.97 ± 9.17		
Bachelor or above	81 (26.56)	47.79 ± 9.21		
Monthly family income (yuan)			*F* = 5.937	0.003
<3000	67 (21.97)	55.02 ± 13.43		
3000~5000	76 (24.92)	51.43 ± 10.05		
>5000	162 (53.11)	49.49 ± 10.40		
Course of diabetes			*t* = −2.465	0.014
<10 years	160 (52.46)	49.69 ± 10.02		
≥10 years	145 (47.54)	52.84 ± 12.26		
Diabetic complication			*t* = −0.602	0.548
Yes	153 (50.16)	49.79 ± 9.59		
No	152 (49.84)	50.63 ± 10.91		
Hypoglycemia in the past year			*t* = 1.770	0.078
Yes	135 (44.26)	52.50 ± 12.54		
No	170 (55.74)	50.15 ± 9.98		
Treatment method			*F* = 4.224	0.006
Oral hypoglycemic agents	120 (39.34)	49.14 ± 9.64		
Insulin	54 (17.70)	50.93 ± 10.74		
Insulin and oral hypoglycemic agents	101 (33.11)	54.27 ± 12.71		
Diet and exercise	30 (9.84)	49.50 ± 10.96		
HbA1c (%)			*t* = −1.991	0.047
<7	104 (34.10)	49.17 ± 9.94		
≥7	201 (65.90)	51.87 ± 11.59		
BMI (kg/m^2^)			*F* = 0.322	0.810
≤18.4	9 (2.95)	51.00 ± 7.52		
18.5~23.9	104 (34.10)	52.06 ± 11.49		
24~27.9	140 (45.90)	50.65 ± 11.28		
≥28	52 (17.05)	50.94 ± 11.28		

**Table 4:** Correlation analysis of trait impulsivity, negative emotions, and EFs (*n* = 305).

	*M*	SD	1	2	3	4	5	6	7	8	9	10
(1) Trait impulsivity	1.77	0.52	1									
(2) Poor self-regulation	1.60	0.66	0.839^∗∗^	1								
(3) Impulsive behavior	1.93	0.61	0.808^∗∗^	0.356^∗∗^	1							
(4) Negative emotions	19.69	15.46	0.396^∗∗^	0.271^∗∗^	0.385^∗∗^	1						
(5) Depression	5.26	5.64	0.400^∗∗^	0.296^∗∗^	0.366^∗∗^	0.899^∗∗^	1					
(6) Anxiety	6.47	5.11	0.337^∗∗^	0.258^∗∗^	0.298^∗∗^	0.882^∗∗^	0.692^∗∗^	1				
(7) Stress	7.95	6.37	0.336^∗∗^	0.189^∗∗^	0.371^∗∗^	0.922^∗∗^	0.740^∗∗^	0.725^∗∗^	1			
(8) GEC	51.19	11.23	0.552^∗∗^	0.381^∗∗^	0.535^∗∗^	0.663^∗∗^	0.629^∗∗^	0.544^∗∗^	0.616^∗∗^	1		
(9) BRI	52.74	11.35	0.530^∗∗^	0.347^∗∗^	0.534^∗∗^	0.665^∗∗^	0.619^∗∗^	0.531^∗∗^	0.639^∗∗^	0.951^∗∗^	1	
(10) MI	50.38	11.20	0.507^∗∗^	0.376^∗∗^	0.462^∗∗^	0.587^∗∗^	0.571^∗∗^	0.489^∗∗^	0.526^∗∗^	0.943^∗∗^	0.833^∗∗^	1

^∗∗^ *p* <0.01.

**Table 5:** Mediating analysis of negative emotions on the relationship between trait impulsivity and BRI and MI (*n* =305).

Paths	Effects	Std. *β*	*β*	SE	*p*	95% CI
Trait impulsivity → BRI	Total effect	0.771	1.431	0.364	<0.001	[0.644, 0.898]
	Direct effect	0.543	1.008	0.375	<0.001	[0.354, 0.750]
Trait impulsivity → negative emotions → BRI	Indirect effect	0.228	0.423	0.096	<0.001	[0.149, 0.332]
Trait impulsivity → MI	Total effect	0.723	1.206	0.276	<0.001	[0.569, 0.855]
	Direct effect	0.494	0.825	0.306	0.007	[0.297, 0.715]
Trait impulsivity → negative emotions → MI	Indirect effect	0.229	0.382	0.090	<0.001	[0.141, 0.336]

## Data Availability

The raw data supporting the conclusions of this article will be made available by the authors without undue reservation.
